# Structural Analysis of Assists in Top Men`s Basketball Teams

**DOI:** 10.5114/jhk/201220

**Published:** 2025-09-23

**Authors:** Uroš Zadnik, Frane Erčulj, Igor Štirn

**Affiliations:** 1 Faculty of Sport, University of Ljubljana, Ljubljana, Slovenia.

**Keywords:** playing positions, game statistics, FIBA, pass

## Abstract

In basketball, an assist is defined as a pass that leads directly to a teammate scoring. Most studies to date have only considered assists as a statistical variable. We decided to take a closer look at the structure of assists using a sample of the top 16 European national teams participating in the 2022 European Championship. We used Synergy Sports technology to collect the data. In a total of 16 matches, 192 players made 640 assists. The data showed that point guards made 40.5% of all assists, while tall players received the most. Additionally, 57.3% of assists were given from the area above the free throw line and 24.3% from the paint. Assists on the move or off the dribble outnumbered assists while standing. Only slightly more than half (53.8%) of the assists were made with both hands, while there were clear differences between air and bounce passes (80.6% compared to 19.4%). The direct involvement of a third player in scoring a basket after an assist was only 12.5%. Surprisingly, winning teams made on average only one more assist than losing teams, which is probably due to the great equality of the competing teams.

## Introduction

In the FIBA statisticians’ manual, an assist is defined as “a pass that leads directly to a team-mate scoring” (FIBA, 2018). A pass to a player inside the paint, who scores from inside the paint and a pass to a player outside the paint, who scores without dribbling, is always considered an assist, as is a pass to a player outside the paint, who scores after one or more dribbles if the shooter does not have to beat his defender. More situations, detailed explanations and examples of all possible situations in which an assist can be made are defined and described in detail.

To be credited with an assist, “teammates must coordinate their movements on the court in such a way that a player gains a positional advantage over the opponent by either deceptively moving without the ball and receiving an accurate pass from a teammate or receiving a pass from a teammate that creates an opening on the court for a score” (Melnick, 2001). Melnick (2001) also stated that making an assist requires game intelligence, coordination, anticipation, timing and exquisite execution, and that it captures the altruistic essence of teamwork. The number of assists is one of the most valuable statistical variables of the game and significantly affects the players final efficiency value (Mikołajec et al., 2021). In addition, it is given additional value by the fact that it is usually one of the variables that make up the so-called double or triple-double, which carries a lot of weight when it comes to evaluating the performance (efficiency) of an individual, especially point guards. It was shown that in the strongest European basketball league (Euroleague), the number of assists increases every year and it approaches the NBA level (Erčulj and Štrumbelj, 2013; Mandić et al., 2019). Some authors presented the number of assists as a quality of teamwork, a measure of the cooperation of two and three players in the search for a tactical solution (Mikołajec et al., 2021).

It has been shown that the number of assists correlates strongly with winning matches. Even in the younger age groups (U-16), winning teams had on average more assists than losing teams, especially in tied matches or comparable teams (Lorenzo et al., 2010). Similar findings were reported for the U-20 age group (Ibáñez et al., 2009); besides successful two-point field goals and defensive rebounds, assists were the most important variables that characterized winning teams. In the regular season matches of the elite Spanish championship (ACB League) (García et al., 2013) and at the international level at the 2007 European Championship (Csataljay et al., 2009), the number of assists was one of the key factors that differentiated winning teams from the losing ones. In contrast, when analysing performance of American NCCA teams (Conte et al., 2018), it was found that a higher number of assists for winning teams was one of the most important indicators of their efficiency, although it did not represent a significant difference between winning and losing teams.

An assist is basically a pass, which is a very important element of the game of basketball and as such has been frequently observed by sports scientists (Quílez-Maimón et al., 2021; Zhang et al., 2018). Passes have therefore been analysed from various points of view: in terms of the technique used (one-handed/two-handed, air pass/bounce pass, dribble pass, etc.), the position or the location on the court where players pass the ball, etc. As for assists, most studies in men`s basketball have considered assists merely as a statistical variables, with various authors limiting themselves to analysing the number of assists in relation to the team or the individual player’s performance. A more detailed analysis of assists in relation to their structure cannot be found in the scientific literature. For both, practice and theory of basketball, there is a lack of information about which types of players make the most assists and which receive the most, in which locations (parts of the court) they occur more or less frequently and how they are executed (from the dribble, in a static position, running), how often a third player is indirectly involved in the action of two players etc. For this reason, we decided to examine the structure of assists in more detail using a sample of the best men`s European national teams. In this way, we aimed to deepen the knowledge and understanding of the importance of this element that gives an additional collective spirit to the game of basketball.

## Methods

### Participants

We analysed the best 16 of 24 teams who participated in the 2022 FIBA European Basketball Championship. Eight bracket matches of the round of 16, four quarter-finals matches, two semi-final matches and the final matches for the third and the first place were included in the analysis. In a total of 16 matches, 192 players made 640 assists, which were further analysed.

### Measures

We analysed assists according to the following criteria:

- the playing position of a player who made the assist (1: point guard (PG), 2: shooting guard (SG), 3: small forward (SF), 4: forward (SF), 5: centre (CE));
- the playing position of a player who received the assist (1: point guard, 2: shooting guard, 3: small forward, 4: forward, 5: centre);
- the location (position on the court) of the player being awarded an assist;
- the way the player moved before receiving an assist (get open, cut to the basket, static position). Cutting was defined as moving to the rim without the ball, getting open as the movement of a player without the ball to receive a pass with the intention of shooting outside the paint, while a static position was defined as a stance of a player on the court who did not move.
- assists performed with or without a dribble;
- assists performed in motion or in a static position;
- the technique of passing according to the use of hands (two-handed, one-handed);
- the technique of passing according to the flight of the ball when assisting (air pass, bounce pass);
- the structure of pairs of cooperating players who gave and received an assist;
- direct involvement of a third player in the assist, defined as a situation in which a third player (not the one who gave the assist, nor the one who scored a point) was involved by setting up a screen and making the pass or the point scored possible. This criteria did not include the so called secondary assists, also labelled “an extra pass” or “an assist to assists” (Supola et al., 2023);
- assists achieved either by the winning or losing teams.

### Design and Procedures

To obtain data, we used Synergy Sports technology (Synergy Sports Technology, San Antonio, Texas, U.S.A., 2013). This software company analyses many of the most important basketball competitions with its professional staff and technology (Božović, 2021; Foteinakis et al., 2024). Among other things, it owns the exclusive rights to provide Euroleague teams with data on all their matches, divided into different categories according to their needs.

The assists included in the analysis were recorded by trained professional employees of the mentioned company, who followed the official instructions of the international basketball organization FIBA (FIBA, 2018). In this way, we obtained video recordings of all assists. The difference in the number of assists recorded live by official competition statisticians and those recorded using Synergy Sports technology was less than 1%.

**Figure 1:**
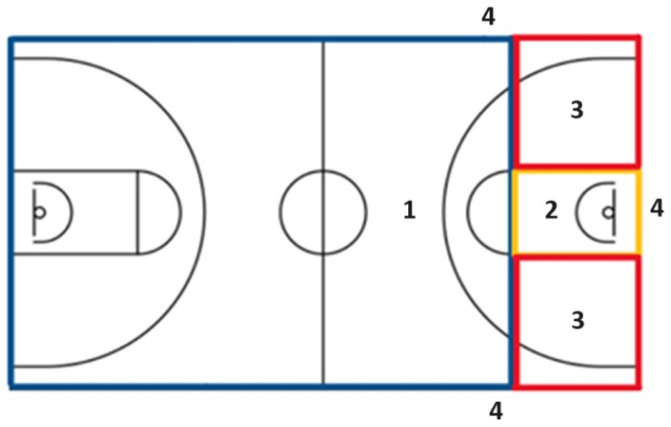
Basketball court divided into 4 zones to determine the location (position on the court) of the player making or receiving an assist. Area 1 (blue): above the free shooting line; Area 2 (yellow): the paint; Area 3 (red): at the both sides of the paint to transverse free shooting line; Area 4: out area, outside side-lines and baseline/end-line

We divided the court area as shown in Figure 1. Zone 1 (the zone inside the blue rectangle) covered the area above the free throw line on the basketball court. Zone 2 (the zone inside the yellow rectangle) defined the area inside the paint and zone 3 (the zone inside the red squares) covered the area below the free throw line and outside the paint. Zone 4 was the space outside the lines of the basketball court (out). We chose these areas because players of various playing positions spent most of their time there. We did not choose to differentiate between the right and the left side of the court, as well as the defensive and the offensive half of the court, because we did not want to compromise the statistical significance with too many variables.

### Statistical Analysis

The obtained data were analysed using the statistical program IBM SPSS. Descriptive statistics and measures of the central tendency and/or measures of dispersion were calculated for the observed variables.

We used an independent *t*-test to compare the average number of assists between the winning and losing teams. Previously, the assumption of normality of distribution (Shapiro-Wilk test) and homogeneity of variances (Levene's test) were checked along with the assumptions of independence of groups.

To analyse differences between playing positions, we used one-way analysis of variance. When determining the differences in scored assists between playing positions, we performed a non-parametric form of the test (Kruskall-Wallis test) due to violated assumptions. When comparing playing positions with regard to the received assists, we performed a non-parametric form of the test (Brown-Forsythe test) due to the violation of the assumption of homogeneity of variances. For additional comparison of particular groups with each other, we used the multiple comparisons test (Games-Howell post hoc test). All data were analyzed at a risk level of 5%.

## Results

**Figure 2:**
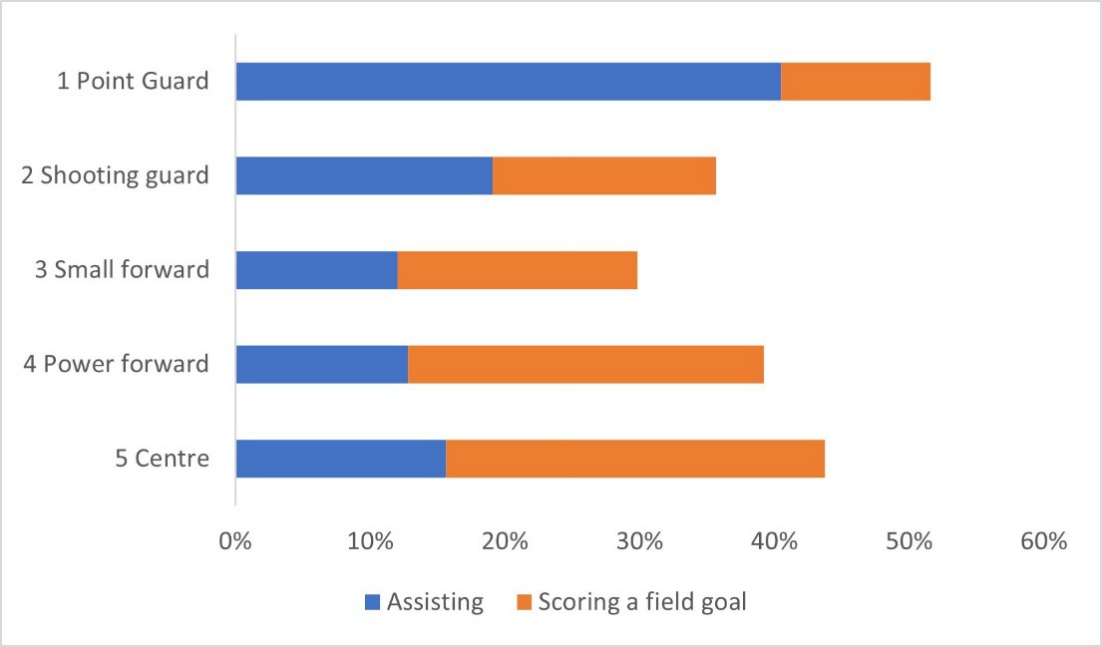
Comparison of made and received assists with regard to the playing position.

Figure 2 shows the comparison of the number of assists made in relation to the playing position of the player who made it (blue bars). Most assists were made by point guards (259 or 40.5% of all assists), followed by shooting guards who made 122 assists, which corresponds to 19.1%. Then there were centres with 100 assists (15.6%) and power forwards with 82 assists (12.8%). The fewest assists (77.12%) were performed by small forwards. A Kruskal-Wallis H-test showed that there were statistically significant differences in the number of assists made between playing positions of players who made these assists (χ2(16) = 42.93, *p* < 0.001) with a mean of 16, 8, 4, 5 and 6 for PG, SG, SF, PF and CE, respectively. Based on post-hoc comparisons (Table 1), it was also found that differences occurred only between point guards (PG) and other players, while no differences were found among SG, SF, PF and CE. The Cohen’s *d* (Table 1) shows predominantly large values, with the exception of the low values for the comparison between SF and PF (*d* = −0.15) and the medium values (*d* = 0.49) for SG-CE and PF-CE (*d* = −0.44). In addition to comparing assists made between playing positions, we were also interested in comparing assists received (Figure 2, orange bars). Centres received the most assists with the number of 180. They were closely followed by power forwards who received 11 less. Small forwards received 114 assists, shooting guards 106 and point guards only 71 in 16 matches. The effect sizes show meaningful correlations between playing positions; only for the SG-SF (*d* = −0.18) and PF-CE (*d* = −0.20) we found low values.

**Table 1:** Differences in made and received assists between playing positions.

		Assists made				Assists received			
Playing position		Cohen's *d*	Lower	Upper	*p*	Cohen's *d*	Lower	Upper	*p*
PG	SG	2.39	1.67	3.11	**0.007**	−1.05	−1.77	−0.33	**0.046**
PG	SF	3.41	2.69	4.13	**0.000**	−1.09	−1.81	−0.37	**0.039**
PG	PF	3.30	2.57	4.02	**0.000**	−2.43	−3.16	−1.71	**0.000**
PG	CE	2.73	2.01	3.45	**0.000**	−2.34	−3.06	−1.61	**0.000**
SG	SF	1.17	0.45	1.89	0.182	−0.18	−0.9	0.54	0.987
SG	PF	1.03	0.31	1.75	0.425	−1.37	−2.09	−0.65	**0.005**
SG	CE	0.49	−0.23	1.21	1.000	−1.43	−2.16	−0.71	**0.003**
SF	PF	−0.15	−0.88	0.57	1.000	−1.09	−1.81	−0.36	**0.033**
SF	CE	−0.58	−1.30	0.15	1.000	−1.18	−1.9	−0.46	**0.018**
PF	CE	−0.44	−1.17	0.28	1.000	−0.2	−0.92	0.53	0.981

PG: point guard, SG: shooting guard, SF: small forward, PF: power forward, CE: centre, p: statistical significance; significant values are in bold

Using ANOVA, we found significant differences between the number of assists received between different playing positions (F(4.75) = 15.03, *p* < 0.001). Post-hoc tests showed that point guards received fewer assists than shooting guards, small forwards, power forwards and centres (Table 1, *p* < 0.05). Shooting guards (*p* = 0.005) and small forwards (*p* = 0.033) received fewer assists than power forwards and centres (*p* = 0.003 and *p* = 0.018, respectively). However, there were no significant differences in the average number of assists received between shooting guards and small forwards (*p* = 0.987) and between power forwards and centres (*p* = 0.981).

**Figure 3:**
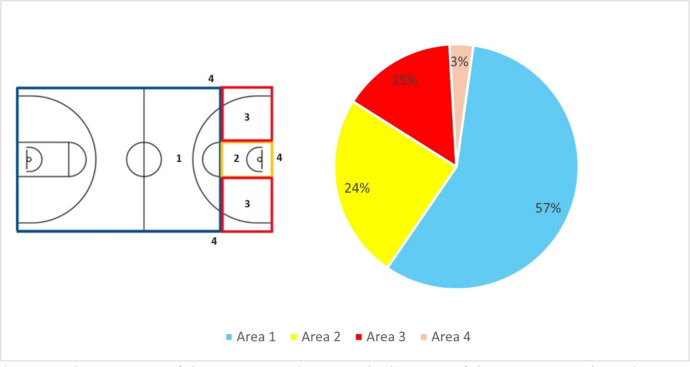
The structure of the assists in relation to the location of the court area where the assistant was positioned when making an assist. Note that colours of the pie chart match the colours of court areas defined in Figure 1

In 367 cases, the assistant was located in the area above the free throw line (area 1) (Figure 3). Assists from the paint (area 2) occurred 156 times, and 97 assists were made in area 3, i.e., from the space below the free-throw line and outside the paint. In 20 cases, the assist was made after the throw-in was performed from out of bounds.

**Figure 4:**
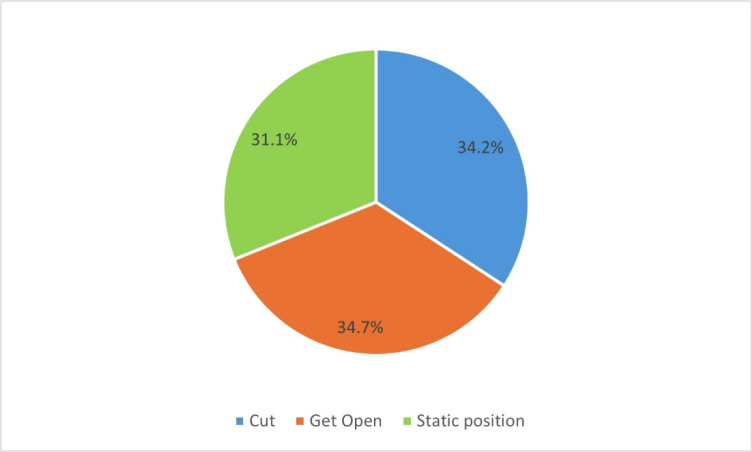
The players' actions before receiving an assist.

Figure 4 shows the actions of players who received the pass and upgraded it into an assist by scoring a basket, before receiving the ball. Players got open 222 times, executed a cut 219 times or simply stood in their spot 199 times.

**Figure 5:**
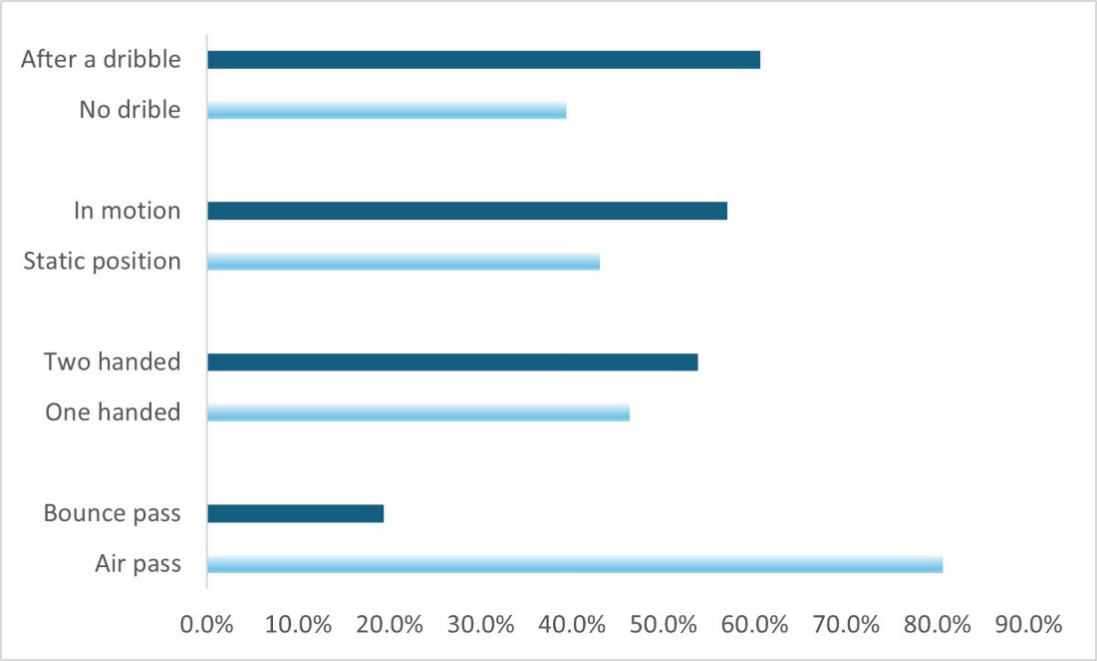
Comparison of assists made in different ways.

As can be seen in Figure 5, almost two thirds of the passes were made after dribbling, predominantly while moving and not being in a static position. Slightly more passes were made two-handed than one-handed, and air passes were far more frequently performed than bounce passes.

**Figure 6:**
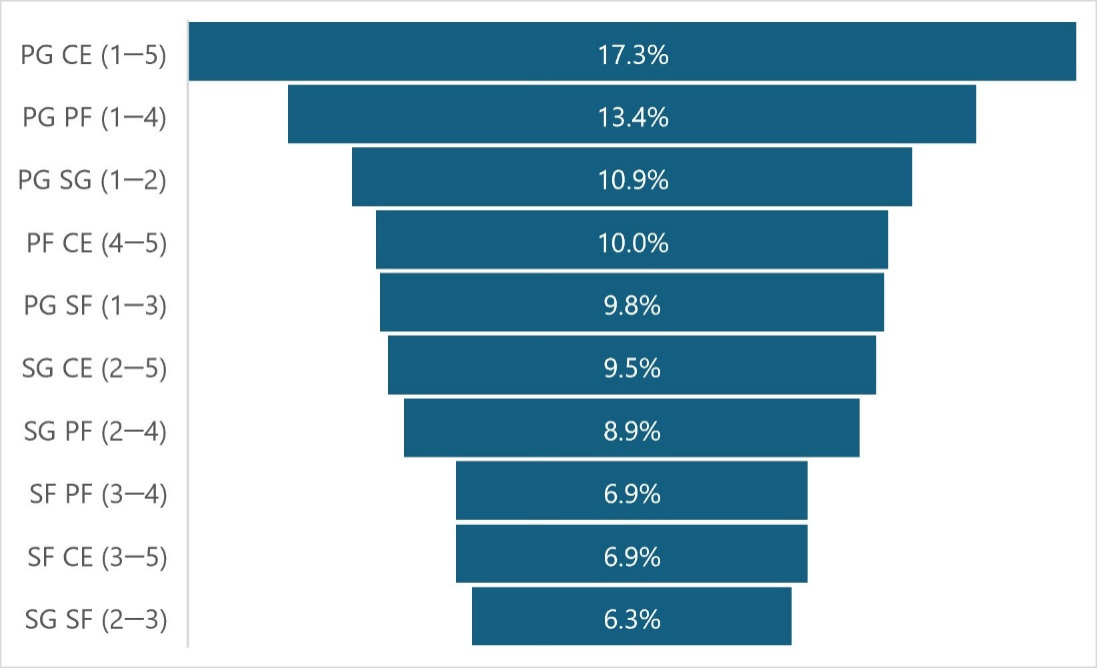
Structure of the cooperation of players who made and received assists in relation to their playing position. PG: point guard; SG: shooting guard; SF: small forward; PF: power forward; CE: centre, 1–5: numeric labels of playing positions

Figure 6 shows the frequency of cooperation between players who assisted and scored a basket. Point guards provided the most assists and collaborated the most with centres and power forwards, while small forwards were involved the least.

We monitored the involvement or direct participation of a third player who was present in fewer cases than we expected. Teams made only 80 assists involving a third player (with making a screen to the player who made or received the assist) and as many as 560 when only two players were directly involved in the assist.

In all 16 games of the final part of the European Championship 2022 combined, winning teams had on average one more assist than losing teams. Winning teams averaged 20.7 assists per game, while losing teams averaged 19.7. There were no significant differences in assists made between winning and losing teams (*p* = 0.541). However, we found that in the most decisive matches, semi-finals and the 3^rd^ and 1^st^ place matches, the winning team made more assists; 20/19 and 32/14 in the semi-finals and 23/21 and 24/20 in the 3^rd^ and the 1^st^ place matches.

## Discussion

Trends show that the number of assists in top basketball teams is increasing (Mandić et al., 2019), suggesting that assists are becoming one of the most important indicators of offensive play effectiveness. Nevertheless, research to date has mainly focused on the technique and frequency of various basketball passes, while assists have not yet been investigated. We analysed 640 assists collected at the national elite level (European Basketball Championship 2022) from different perspectives: in terms of the position of the player who made and received an assist, in terms of the location on the court of the assistant and the action of the player before he received the assist. We also analysed the technique of executing a pass, taking into account ball handling before the pass. Finally, we examined in detail the structure of cooperation between players from different positions and also looked for the direct involvement of a third player.

Surprisingly, the latter was observed less frequently than expected, i.e., only in 12.5% of all detected assists. The low percentage of the third player involvement could be mainly due to the fact that the defensive play of the best European teams was at a very high performance level. They were so well prepared for the opponent's offence that offensive teams rarely got open shots. The relatively low percentage of the third player involvement is also due to the frequent use of individual offensive actions (1-on-1 play) and, above all, the “pick and roll” play. The short roll option was also used and taken into account.

Even more surprising was the fact that in our research, winning teams averaged only one more assist than losing teams (20.7 vs. 19.7 assists per match). This is in contrast to previous research that found a strong correlation between the number of assists and winning matches in all age categories (Ibáñez et al., 2009; Lorenzo et al., 2010), which was even more pronounced in men than in juniors (Csataljay et al., 2009; García et al., 2013; Sampaio et al., 2004). At the highest performance level, in the NBA, there was a strong positive correlation between assists and win/loss records, suggesting that teamwork was crucial for success (Melnick, 2001). Opposite results were reported by Conte et al. (2018) who found that a higher number of assists for winning teams in the NCAA was one of the most important indicators of their efficiency, yet it did not represent a significant difference between winning and losing teams. It has been speculated that this could be due to the style of play in the NCAA league, which unlike in Europe, focuses on a more physical game with a lot of running and 1-on-1 play. However, it is interesting to note that in our study, winning teams dominated in this element in the semi-final, third-place and first-place matches. This could be an indication of the greater importance of assists in more important and decisive matches. Yet another perspective, i.e., scoring as many assists as possible, does not necessarily mean winning the match, but is simply a mandatory norm of playing at the highest performance level.

On the other hand, the rest of the data we collected were largely in line with our expectations. Point guards, sometimes referred to as playmakers, contributed more than 40% of all assists (Figure 2, blue bars), while tall players (PF, CE) stood out in assists received (Figure 2, red bars). This was predictable, as the point guards’ role is the creation and dictation of the play of the other players—playmaking. This is consistent with the findings of Mikołajec et al. (2021) who showed that the number of assists significantly affected the final efficiency score of point guards, and Erčulj et al. (2016b) who found that more than half of all passes in Euroleague 2013 best four teams were made by guards, as many as small forwards and centres combined. When analysing the strongest Spanish and Portuguese leagues, as well as the NBA, it was also found when looking at assists (not passes) that guards made the most assists, even more than small forwards and centres combined (Sampaio et al., 2004).

Playmakers were therefore primarily responsible for giving assists and not receiving them. On the other hand, tall players (centres and power forwards) were the ones who tried to get the ball as close to the rim as possible and score a basket. Tall players are most often involved in a “pick-and-roll” play, meaning that they set up a screen to a teammate (pick) and then move toward the rim (roll), which is another way to get an assist. As the name suggests, shooting guards are usually good shooters, but in many cases, they cooperate with or even substitute the point guard in organizing the play and therefore also score a considerable number of assists. Small forwards are the least involved in setting up plays, i.e., they usually do not make assists except in rare situations such as “extra passes”. However, they receive about the same number of assists as shooting guards and mainly carry out offensive actions known as “catch and shoot”.

Most assists (57.3%) were made from the area above the free throw line (area 1), where point guards spend most of their time moving with the ball. Assists from area 2 (the paint) accounted for 24% of all assists and were mostly the result of passes made by guard players after penetrating below the rim. We found no dominant way of movement prior to receiving an assist. All three ways of ball reception we analysed (getting open, cutting or standing still) worked well for players who received an assist and were fairly balanced, each accounting for about one third of all assists. Assists performed while moving predominated (57%) compared to assists performed while standing (43%) (Figure 5). Assists made on the move could be due to the offense's implementation of its main objective, which is to break through the first line of defense and create a drive and kick option by the ball handler. The so-called “pick and roll” or “pick and pop” plays are often implemented. In this type of play, one player dribbles the ball while the other sets up a screen and then runs (rolls) toward or away from the rim into open space to potentially get an assist.

Another reason for the relatively high proportion of assists performed in motion is the frequent one-on-one play, where players without the ball make space for the player with the ball to make it easier for him to play one-on-one. Assists made while being in a static position are often called the secondary assists, where the player who receives the ball “extends” the pass after the initial pass to a teammate (i.e., “extra pass”) who then takes a clear shot. Secondary assists are often overlooked; however, they play an important role in creating space and improving shot quality, and can serve as an indicator of effective ball movement. It has also been found that teams with a higher number of secondary assists in the NBA more easily get open, unobstructed shots (Supola et al., 2022).

The reason for the higher proportion of assists performed after a dribble (60.6% dribble vs. 39.4% no dribble) is that this type of a pass is faster and less predictable than passes while standing still. In addition, dribble passes are often a result of aggressive defensive play which puts a lot of pressure on the player who is standing on the spot or has caught the ball and stops after dribbling, making the pass impossible. Of course, dribble passes are technically more demanding, thus it is important that basketball players include complex drills in their training, where they can practise this way of passing the ball and, consequently, assists. More than half (52.5%) of the assists observed were made using dribble passes when players were also in motion, whereas in 223 cases (34.8%), assists were made without a dribble, while simply standing in a static position. Less typical were the combinations where the player passed the ball while dribbling on the spot (8.1%) and when in movement, but not dribbling (less than 5%). Out of a total of 640 assists, 344 were performed using both hands, which represents 53.8% of all assists. This is in line with Izzo and Russo (2011) and Erčulj et al. (2016b) who reported that the best teams in the Euroleague 2013 executed an average of 268 passes per match, with passes with both hands being used most frequently (more than 60%). Different passing techniques with one hand occurred in less than 40% of passes, with passes with the dominant hand occurring about three times as often as passes with the non-dominant hand. Our data also showed that players most often used double-handed passes from in front of the chest or over the head. This is consistent with the finding that chest passes were the most common and successful assist technique (Izzo and Russo, 2011). There were 296 one-handed passes for assists, which is 46.75% of the total. The higher proportion of two-handed passes did not surprise us; in fact, we expected a slightly higher proportion of these types of passes, as previous research found over 60% two-handed passes (in general) for the best Euroleague teams (Erčulj et al., 2016b) and junior teams (U16) (Erčulj et al., 2016a). The relatively high proportion of one-handed assists is primarily attributed to the aggressiveness of the defensive play.

We found clear differences when comparing the number of air passes and bounce passes for assists. In the vast majority of cases (80.6%), assists were made as direct (air) passes, and in less than 20% of assists, bounce passes were used. These results also correspond to earlier findings by Izzo and Russo (2011) who analysed top Italian games, some NCAA and NBA games, and by Erčulj et al. (2016b) who examined passing techniques in Euroleague games.

According to Trninić et al. (2002), the ability to assist is influenced by information processing, energy expenditure and socio-motor interaction. Therefore, we also took a closer look at the cooperation patterns between the players (Figure 6). Most cooperation took place between point guards and centres as well as point guards and power forwards. Small forwards were the least involved in the cooperation between assistants and scorers with less than 7%. In other words, they did not give or receive many assists. Interestingly, professional basketball players have been reported to show direct reciprocity in assist patterns, with higher likelihood of returning assists to teammates who have previously assisted them, especially within a short period of time (Willer et al., 2012). It has also been shown that the number of assists (and some other statistical variables, such as the free throw percentage) increases over the course of a player`s career, favouring experienced players in the decisive moments of the game (Ibáñez et al., 2008; Lorenzo et al., 2019). In terms of practice, it has been recently observed that larger game formats are associated with significantly more effective or ineffective passing, even in unbalanced situations (5 vs. 4 or 4 vs. 3 as opposed to 3 vs. 2 or 2 vs. 1, which are often practised in small-sided games), and appear to be better suited to promote passing (de Souza et al., 2024).

## Conclusions

This study analysed assists in detail and thus, it provides a good insight into their structure in top European basketball teams. Our data support previous findings on the importance of technical (biomechanical) and physical conditioning aspects of passing (Maimón et al., 2020). They highlight the importance of assists and passing patterns in basketball and emphasise their role in team success and player interaction on the court.

A limitation of our study was the small number of analysed matches and consequently the number of assists taken into consideration. On the one hand, a larger number of matches would not represent the highest quality matches (only the bracket matches in a round of 16 teams were analysed), on the other hand, a larger number of collected data would allow us to consider some new variables, such as more court areas. If we were to repeat this study, we would probably opt for an additional sector: the centre line of the court (defensive half of the court), especially in the context of assists made in the transition phase, and perhaps the left and the right side of the court. In addition, it would also be reasonable to consider extra passes, as it has been shown that they can indicate better teamwork (Supola et al., 2023). Another possibility for further studies is to investigate this topic in women and in younger age groups.

In the future, statistical data could be enriched with data obtained using passing quality assessment tools based on accuracy, execution time and pattern variability (Quílez-Maimón et al., 2021). Using the data obtained, basketball coaches and other experts in the field can more easily plan training to improve their team tactics, i.e., the development of effective offensive and defensive plays as well as situational passing game training.
